# Safety and Feasibility of Deep Sedation With a Tulip Airway and Jaw Elevation Device During Electrophysiology Procedures: A Single‐Center Experience of 647 Consecutive Cases

**DOI:** 10.1002/joa3.70323

**Published:** 2026-03-25

**Authors:** Kotaro Tsuruta, Kensuke Yokoi, Makoto Edayoshi, Kodai Shinzato, Kana Nakashima, Takanori Yamaguchi

**Affiliations:** ^1^ Department of Cardiovascular Medicine Saga University Saga Japan

**Keywords:** atrial fibrillation, catheter ablation, left atrial appendage closure, sedation

## Abstract

**Background:**

Safe and stable deep sedation is essential for electrophysiology procedures. We developed a propofol‐free deep sedation protocol using the Tulip airway and a jaw elevation device (JED).

**Methods:**

This single‐center retrospective study analyzed 647 consecutive patients without an anticipated difficult airway (634 atrial fibrillation ablation; 13 left atrial appendage closure) who underwent deep sedation. Endpoints included procedural completion, conversion to intubation, sedation discontinuation, and procedure‐related complications.

**Results:**

All procedures were completed. One (0.2%) patient required intubation, and 3 (0.5%) experienced procedure‐related complications.

**Conclusion:**

Deep sedation with the Tulip airway and JED was safe and feasible in selected lower‐risk patients.

## Graphical abstract

A propofol‐free deep sedation protocol using a Tulip airway and jaw elevation device was safe and feasible in 647 selected electrophysiology procedures, with a low incidence of sedation‐related complications.

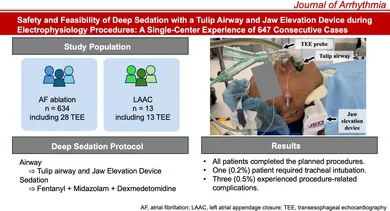

## 1 Introduction

Patient safety and procedural stability through optimal anesthesia or sedation are essential for successful electrophysiology procedures, such as catheter ablation and percutaneous left atrial appendage closure (LAAC) [1]. However, owing to a shortage of anesthesiologists [2], anesthesiologist‐administered general anesthesia is not readily available in Japan. Consequently, cardiologist‐led deep sedation, often using propofol and airway devices, has been widely adopted [3], despite the off‐label use of propofol for sedation. Although the i‐gel (Intersurgical, UK) is commonly used in Japan [4], its design limits oropharyngeal space, making transesophageal echocardiography (TEE) impractical and thereby precluding TEE‐guided procedures, such as LAAC. Therefore, we developed an alternative propofol‐free deep‐sedation strategy that allows TEE when necessary. This study evaluated the safety and feasibility of this approach using the Tulip airway (Age of Aquarius, UK) and a jaw elevation device (JED, Hypnoz Therapeutic Devices, CA, USA) in a single‐center cohort of 647 consecutive cases.

## 2 Methods

### 2.1 Study Population

This single‐center retrospective study was based on ongoing prospective observational studies of atrial biopsy in patients with atrial fibrillation (AF), including 877 patients undergoing AF catheter ablation and 50 patients undergoing LAAC between June 2020 and November 2025 (Supporting Information S1). Patients who underwent the procedures at other institutions (*n* = 11) or under general anesthesia (AF ablation, *n* = 232; LAAC, *n* = 37) were excluded. The remaining 647 patients underwent procedures under deep sedation using a Tulip airway and JED. Procedures were performed under TEE guidance in 41 (6%) patients, comprising 28 atrial biopsies and 13 LAAC procedures.

### 2.2 Sedation Protocol

Patients at high risk for deep sedation—such as those with an anticipated difficult airway (including limited mouth opening < 3 cm); obesity (body mass index ≥ 30 kg/m^2^); diseases of the neck, upper respiratory tract, or upper alimentary tract; severe lung disease; severe left ventricular dysfunction (ejection fraction < 30%); and an American Society of Anesthesiologists physical status classification of ≥ 3—underwent AF ablation under anesthesiologists‐administered general anesthesia.

**FIGURE 1:**
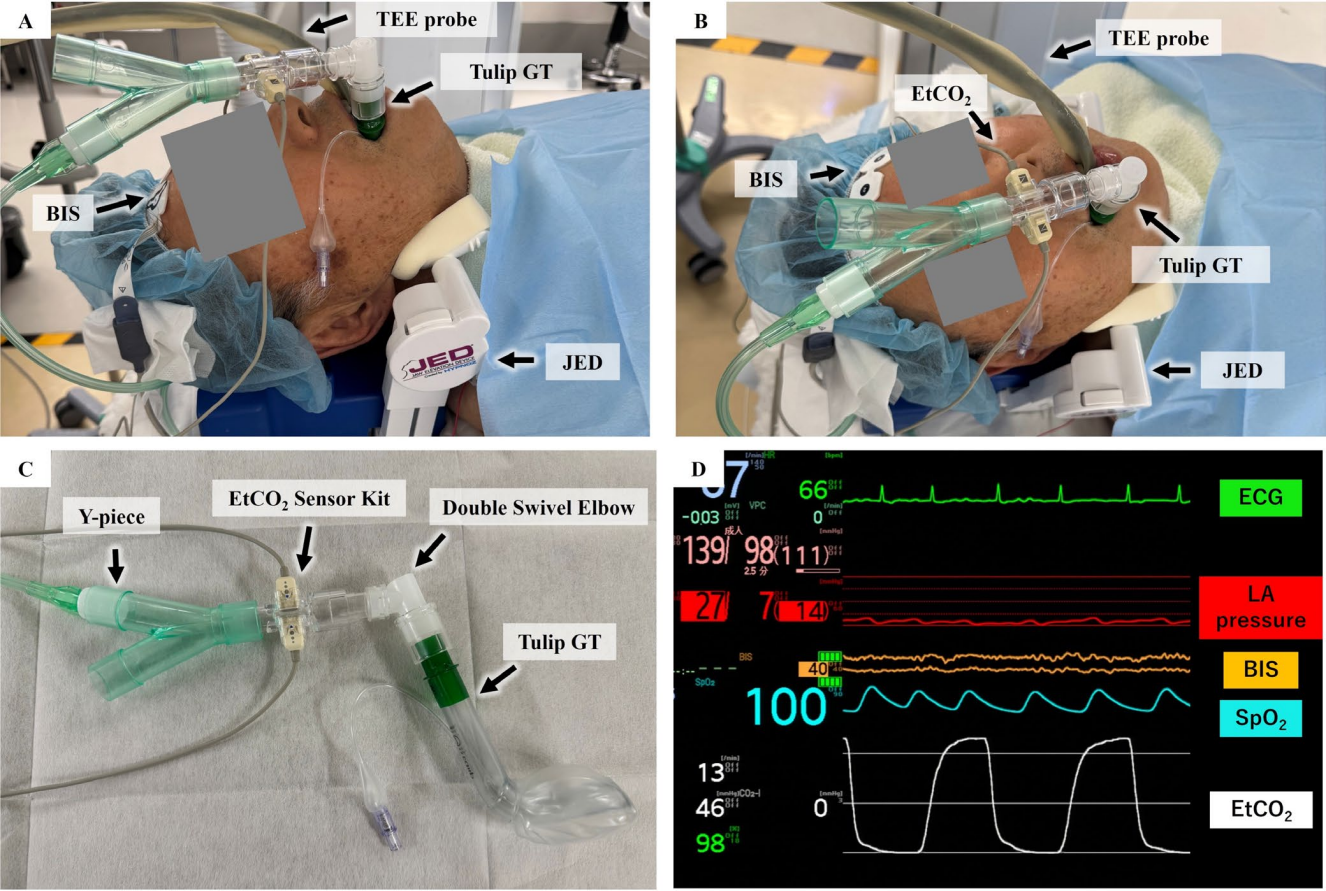
Integration of the Tulip airway and a jaw elevation device (JED). (A, B) Side and front anterior views of the patient setup. Airway patency is maintained using the Tulip airway, which enables the simultaneous use of transesophageal echocardiography (TEE). The JED provides consistent mechanical jaw thrust to prevent upper airway obstruction. (C) Configuration of the ventilation circuit. The system integrates the Tulip GT (Age of Aquarius, UK), a double swivel elbow (Intersurgical Ltd., UK), a CO_2_ sensor kit (Nihon Kohden, Japan), and a Y‐piece (Intersurgical Ltd.). (D) Representative intraprocedural monitoring during left atrial appendage closure. Continuous monitoring of vital signs, the bispectral index (BIS) for sedation depth, and end‐tidal carbon dioxide (EtCO_2_) waveforms ensures hemodynamic stability, an appropriate level of anesthesia, and adequate ventilation throughout the procedure.

Deep sedation, defined as a level sufficient to permit external cardioversion without patient movement or awareness, was administered and supervised by cardiologists experienced in airway management. Anesthesiology personnel were not routinely involved. All patients were preoxygenated with 5 L/min of oxygen via a facemask, and routine monitoring, including oxygen saturation, end‐tidal CO_2_ (EtCO_2_), and bispectral index (BIS) monitoring, was performed. Sedation was initiated with an intravenous bolus of fentanyl (50 μg) and midazolam (0.1 mg/kg), followed by a dexmedetomidine loading dose (6 μg/kg/h for 10 min) and maintenance infusion (0.7 μg/kg/h). Supplemental thiamylal sodium (50–75 mg) was administered just before airway and/or TEE probe insertion. Once the patient was unresponsive, the Tulip GT (size 2/green color) was inserted, and its cuff was inflated with 20–40 mL of air. A JED was also used to maintain airway patency as much as possible (Figure 1). When necessary, the TEE probe was inserted through the piriform fossa under fluoroscopic guidance to minimize stimulation of the glottis (Video S1). Oxygen was delivered via the airway device, and EtCO_2_ was continuously monitored. The target BIS range was 40–60. During the procedure, fentanyl (50 μg) was additionally administered every 30–40 min and titrated to maintain a respiratory rate of ≥ 10 breaths/min. Thiamylal (50–75 mg) was also administered as needed during radiofrequency energy delivery and patient movements. When oxygen saturation fell below 90% for > 1 min, manual ventilation was performed using a bag‐valve mask.

### 2.3 Endpoint

The endpoints were feasibility and safety, assessed by the procedural completion rate; sedation‐related complications, defined as hypoxia requiring conversion to endotracheal intubation or discontinuation of sedation, or laryngospasm persisting for > 1 min; procedure‐related complications excluding vascular access‐site complications; and procedure time, defined as the interval from sedation initiation to sheath removal.

## 3 Results

The 647 patients (mean age, 67 ± 11 years; female, 28%) underwent AF ablation (*n* = 634) or LAAC (*n* = 13). Patient characteristics are shown in Table 1. All patients completed the planned procedures. Oxygen saturation decreased to < 90% during sedation induction in 60 patients (9%). In 8 patients (1%), desaturation persisted for > 1 min and required bag‐valve mask ventilation; all episodes occurred during induction. Among these, 1 patient (0.2%) required tracheal intubation because of respiratory deterioration, possibly related to underlying chronic obstructive pulmonary disease, and was classified as having a sedation‐related complication. Procedure‐related complications occurred in three patients (cardiac tamponade, *n* = 2; phrenic nerve injury, *n* = 1). The mean procedure times were 148 ± 37 and 65 ± 24 min for AF ablation and LAAC, respectively. Hypotension requiring vasopressor administration occurred in 145 patients (22%). Other data regarding sedation are provided in Table 2.

**TABLE 1:** Patient characteristics.

Category	Variable	Value
Demographics	Age (years)	67 ± 11
	Female, *n* (%)	183 (28%)
	BMI (kg/m^2^)	24.5 ± 3.6
Comorbidities	CHADS_2_ score	1.4 ± 1.2
	CHA_2_DS_2_‐VASc score	2.3 ± 1.6
	eGFR (mL/min/1.73 m^2^)	68 ± 19
	Hemodialysis, *n* (%)	13 (2.0%)
	Hypertension, *n* (%)	379 (59%)
	Type 2 diabetes mellitus, *n* (%)	109 (17%)
	Congestive heart failure, *n* (%)	140 (22%)
	Prior stroke, *n* (%)	60 (9%)
	Chronic obstructive pulmonary disease, *n* (%)	13 (2%)
	Obstructive sleep apnea, *n* (%)	20 (3%)
Arrhythmia type	Paroxysmal AF, *n* (%)	292 (45%)
	Persistent AF, *n* (%)	355 (55%)
Echocardiography	LA diameter (mm)	41 ± 6
	LA volume/BSA (mL/m^2^)	44 ± 16
	Left ventricular ejection fraction (%)	59 ± 12

*Note:* Categorical variables are presented as numbers (percentages) and continuous variables as means ± standard deviations.

Abbreviations: AF, atrial fibrillation; BMI, body mass index; BSA body, surface area; eGFR, estimated glomerular filtration rate; LA, left atrium.

**TABLE 2:** Procedural data.

Variable	Value
*Procedure type, n (%)*	
AF ablation	634 (98%)
LAAC	13 (2%)
*Imaging guidance, n (%)*	
Transesophageal echocardiography	41 (6%)
Intracardiac echocardiography	606 (94%)
Procedure time (AF ablation)	148 ± 37
Procedure time (LAAC)	65 ± 24
*Sedative and analgesic dosages (total)*	
Midazolam (mg)	7.2 ± 1.5
Fentanyl (μg)	140 ± 50
Dexmedetomidine (μg)	148 ± 39

*Note:* Categorical variables are presented as numbers (percentages) and continuous variables as means ± standard deviations.

Abbreviations: AF, atrial fibrillation; LAAC, left atrial appendage closure.

## 4 Discussion

This study demonstrates that our propofol‐free deep sedation protocol using the Tulip airway and a JED is safe and feasible in a carefully selected, lower‐risk population undergoing electrophysiology procedures. Specifically, patients with anticipated difficult airway, obesity, or higher anesthetic risk were excluded. Therefore, the present findings should be interpreted within the context of this selected population. Previous studies have reported the clinical usefulness of the JED and Tulip airway individually [5, 6]. In this study, their combined use achieved a high procedural completion rate, supporting the procedural stability in this low‐risk population. Notably, TEE was successfully performed in 41 patients, indicating this sedation strategy may serve as a practical alternative to general anesthesia for selected TEE‐guided procedures, such as LAAC. Furthermore, the JED provides a hands‐free mechanical jaw thrust. This may reduce the need for additional personnel and potentially decrease staff radiation exposure during fluoroscopy‐guided procedures.

## 5 Limitations

Given the single‐center retrospective design, the findings are applicable only to a selected lower‐risk population, as patients with anticipated difficult airway, obesity, or other high‐risk features were managed under anesthesiologist‐administered general anesthesia. Although TEE‐guided procedures were feasible in 41 cases, larger multicenter prospective studies are needed to confirm the safety and generalizability of this protocol.

## 6 Conclusion

The proposed deep sedation protocol using the Tulip airway and a JED is safe and feasible for electrophysiology procedures in selected patients, and may serve as an alternative to general anesthesia for certain TEE‐guided procedures.

## Funding

This work was supported by Japan Society for the Promotion of Science (JSPS) KAKENHI Grant‐in‐Aid for Scientific Research (A) (JP22H00471); Japan Society for the Promotion of Science (JSPS) KAKENHI Grant‐in‐Aid for Scientific Research (C) (JP21K08056); and Japan Agency for Medical Research and Development (JP22ek0210164, JP23ek0210164, JP18km0405209, JP23tm0724607, JP24ek0109755).

## Ethics Statement

This study was approved by the Ethics Committee of Saga University Hospital (approval reference numbers: 20200101 for HEAL‐AF, 20200901 for HEAL‐AF2, 202200401 for FUTURE‐AF‐S, 20220401 for HEAL‐AF3, 20240804 for HEAL‐AF4, and 20230501 for LEARNMORE studies).

## Conflicts of Interest

Takanori Yamaguchi received honoraria from Abbott Medical Japan and Medtronic Japan. Kensuke Yokoi, Kana Nakashima, and Takanori Yamaguchi are also affiliated with the Department of Advanced Management of Cardiac Arrhythmia, Saga University, sponsored by Abbott Medical Japan, Nihon Kohden Corporation, Medtronic Japan, Japan Lifeline, Boston Scientific Japan, and Fides‐ONE Corporation. The remaining authors declare that they have no conflicts of interest.

## Supporting information


**Data S1:** joa370323‐sup‐0001‐Supinfo1.docx.

**Video S1:** Insertion of the transesophageal echocardiography (TEE) probe through the piriform fossa under fluoroscopic guidance to minimize stimulation of the glottis.

## Data Availability

The data that support the findings of this study are available on request from the corresponding author. The data are not publicly available due to privacy or ethical restrictions.

## References

[1] S. Tzeis , E. P. Gerstenfeld , J. Kalman , et al., “European Heart Rhythm Association/Heart Rhythm Society/Asia Pacific Heart Rhythm Society/Latin American Heart Rhythm Society Expert Consensus Statement on Catheter and Surgical Ablation of Atrial Fibrillation,” Europace 26 (2024): euae043. 38587017 10.1093/europace/euae043 PMC11000153

[2] M. Tamai , S. Kojima , Y. Baba , and K. Kurahashi , “Variabilities and Contentions in Anesthesiologists' Perspectives on Japanese Perianesthesia Nurses: A Qualitative Study,” PLoS One 19 (2024): e0313158. 39739952 10.1371/journal.pone.0313158 PMC11687901

[3] K. Inoue , Y. Murakawa , A. Nogami , et al., “Current Status of Catheter Ablation for Atrial Fibrillation‐Updated Summary of the Japanese Catheter Ablation Registry of Atrial Fibrillation (J‐CARAF),” Circulation Journal 2014, no. 78 (2014): 1112–1120. 10.1253/circj.cj-13-1179 24632790

[4] T. Yamaguchi , Y. Shimakawa , S. Mitsumizo , et al., “Feasibility of Total Intravenous Anesthesia by Cardiologists With the Support of Anesthesiologists During Catheter Ablation of Atrial Fibrillation,” Journal of Cardiology 72 (2018): 19–25. 29338895 10.1016/j.jjcc.2017.12.008

[5] A. Shaikh , P. N. Robinson , and M. Hasan , “The Tulip GT Airway Versus the Facemask and Guedel Airway: A Randomised, Controlled, Cross‐Over Study by Basic Life Support‐Trained Airway Providers in Anaesthetised Patients,” Anaesthesia 71 (2016): 315–319. 26684684 10.1111/anae.13328

[6] S. H. Lee , J. S. Jeong , J. Jang , et al., “Comparison of Jaw Elevation Device vs. Conventional Airway Assist During Sedation in Chronic Kidney Diseases Undergoing Arteriovenous Fistula Surgery: A Randomized Controlled Trial,” Journal of Clinical Medicine 10 (2021): 2280. 34074066 10.3390/jcm10112280 PMC8197371

