# Supplementary material for: Safety and Feasibility of Deep Sedation With a Tulip Airway and Jaw Elevation Device During Electrophysiology Procedures: A Single‐Center Experience of 647 Consecutive Cases
Source: J Arrhythm. 2026 Mar 25;42(2):e70323. doi: 10.1002/joa3.70323 (PMC13531356; doi:10.1002/joa3.70323)
Supplement: Supplementary file 1 [file JOA3-42-e70323-s002.docx]

**Supplementary Materials**

**Safety and Feasibility of Deep Sedation with a Tulip Airway and Jaw Elevation Device during Electrophysiology Procedures: A Single-Center Experience of 647 Consecutive Cases**

**Kotaro Tsuruta, Kensuke Yokoi, et al.**

**Atrial Biopsy Studies**

Patients were enrolled from the following observational studies. All studies were approved by the Ethics Committee of the Saga University Hospital.

1. Histological Evaluation of Atrial Fibrillation Substrate Based on Atrial Septum Biopsy (HEAL-AF Study); n = 103; approval reference number: 20200101

2. Histological Evaluation of Atrial Fibrillation Substrate Based on Atrial Septum Biopsy 2 (HEAL-AF Study 2); n = 300; approval reference number: 20200901

3. Histological Evaluation of Atrial Fibrillation Substrate Based on Atrial Septum Biopsy 3 (HEAL-AF Study 3); n = 20; approval reference number: 20220401

4. Histological Evaluation of Atrial Fibrillation Substrate Based on Atrial Septum Biopsy 4 (HEAL-AF Study 4); n = 54; approval reference number: 20240804

5. Follow-up Study of Patients Undergoing Catheter Ablation for Atrial Fibrillation: Evaluation of Long-term Outcomes and Predictive Factors Based on Genetic Predisposition—Subgroup Analysis in Patients with Atrial Biopsy and Proteomics (FUTURE-AF-S); n = 400; approval reference number: 20220401

6. Left Atrial Appendage Occlusion and Histological Assessment of Atrial Substrate in Patients with Atrial Fibrillation (LEARNMORE); n = 50; approval reference number: 202300501

**Details of Atrial Biopsy**

Atrial biopsy was performed during atrial fibrillation catheter ablation or left atrial appendage closure under intracardiac echocardiography or transesophageal echocardiography, in addition to fluoroscopy guidance. Up to five biopsy samples were obtained per procedure from the posterior portion of the limbus of the fossa ovalis in the right atrium. Detailed procedural methods have been described previously [1–5]. Atrial biopsy was completed within 15 minutes in all patients.

1. Otsubo T, Shinzato K, Yamaguchi T, Nakashima K, Takahashi Y, Tsuruta K, et al. Feasibility and safety of atrial biopsy - evidence from 1,000 cases. Circ Rep. 2025;7:764-773.

2. Yamaguchi T, Otsubo T, Takahashi Y, Nakashima K, Fukui A, Hirota K, et al. Atrial structural remodeling in patients with atrial fibrillation is a diffuse fibrotic process: evidence from high-density voltage mapping and atrial biopsy. J Am Heart Assoc. 2022;11:e024521.

3. Takahashi Y, Yamaguchi T, Otsubo T, Nakashima K, Shinzato K, Osako R, et al. Histological validation of atrial structural remodelling in patients with atrial fibrillation. Eur Heart J. 2023;44:3339-3353.

4. Shinzato K, Takahashi Y, Yamaguchi T, Otsubo T, Nakashima K, Yoshioka G, et al. Atrial amyloidosis identified by biopsy in atrial fibrillation: prevalence and clinical presentation. Eur Heart J. 2025;46:3437-3449.

5. Nakashima K, Yamaguchi T, Takahashi Y, Otsubo T, Shichida S, Osako R, et al. Sex differences in atrial fibrillation-related atrial remodelling assessed by electroanatomic mapping and biopsy. Eur Heart J. 2026;47:217-231.

**Supplementary Video**

Insertion of the transesophageal echocardiography (TEE) probe through the piriform fossa under fluoroscopic guidance to minimize stimulation of the glottis.
